# Supplementary material for: Reformulation of Top-Selling Processed and Ultra-Processed Foods and Beverages in the Peruvian Food Supply after Front-of-Package Warning Label Policy
Source: Int J Environ Res Public Health. 2022 Dec 27;20(1):424. doi: 10.3390/ijerph20010424 (PMC9819345; doi:10.3390/ijerph20010424)
Supplement: Supplementary file 1 [file ijerph-20-00424-s001.zip › ijerph-2043794-supplementary.pdf]

Table S1. Foods and beverages categories included in the study

Group	Study categories	Descriptions/Examples
Foods	Bread and Bakery Products	Pre-packaged foods made primarily of wheat flour with added additives / Cookies, muffins
	Cereals	Flavored wheat flakes and bars / Breakfast cereal, cereal bar
	Candies and Desserts	Sweets, chocolates powered desserts that are not grain based/ Candies, chocolates, jelly
	Ice creams	Desserts based on milk / Milk based ice cream, ice cream-based desserts
	Prepared, Ready to Eat Meals	Ready-to-eat preparations / Instant soups, creamy vegetable soup
	Meat and Eggs	Processed meat with added additives / Ham, hot dog
	Dairy Based products	Processed food based on milk / Cream cheese, yogurt
	Sauces, Spreads and Oils	Sauces that accompany the preparations, salad dressings and margarine / Ketchup, mustard, chili sauces, salad dressings, mayonnaise, margarine
	Snacks	Salty snacks / Potatoes chips, pretzels, cheese snacks, puffed snacks, corn based chips
Beverages	Juice, nectars and flavored juice drinks	Flavored and sweetened beverages with juice or artificial flavored joice / Flavored juice, nectars
	Carbonated drinks	Carbonated beverages / Sodas, sparkling flavored waters
	Sports drinks	Rehydrating drinks / Sport drinks
	Teas	Tea based beverage / Ready to drink tea
	Milk, dairy drinks	Flavored milk drinks / Drinkable yogurt, chocolate milk, flavored milk
	Powdered milk flavors	Soluble dairy powders / Milk prepared with flavored powder

Table S2. Terms used for the identification of non-nutritive sweeteners and polyols

**Terms (in English and Spanish)**	Acesulfame potassium, acesulfame de potasio, acesulfame k, acesulfame potasico, acesulfamo k, acesulfamo de potasio, acido cordicepico, acido ciclamico, ciclamic acid, adonitol, advantamo, advantame, aspartil fenilalanina metil ester, alitame, almindo hidrolizado hidrogenado, arabitol, aspartame, aspartamo, ciclamato de calcio, calcium cyclamate, ciclamato de sodio, sodium ciclamate, cordycepic acid, d-glucitol, d-glucitol syrup, d-maltitol, d-mannitol, d-sorbitol, edulcorante, edulcorantes, edulcorantes artificiales, endulzante, eritritol, erythritol, eritrite, estevia, extracto puro de Stevia, extracto puro de stevia rebaudiana, fructosa y stevia, fucitol, glucósidos de esteviol, extracto seco de estevia, estevioglicósidos, rebaudosido, steviol glycosides, rebaudiosido a, galactitol, glicerol, glucitol, glucosido de steviol e 960, glycerin, glicerina, glycerine, granulado de Stevia, hydrogenated glucose syrup, hydrogenated isomaltulose, hydrogenated maltitol, hydrogenated starch hydrolysates, hydrogenated starch hydrolysate, hydrogenated starchhydrolysates, hydrogenatedstarch hydrolysates, iditol, inositol, isomalt, isomalt (e 953), isomalta, isomaltitol, isomaltol, isomaltulosa hidrogenada, jarabe de d-glucitol, jarabe de glucosa hidrogenada, jarabe de maltitol, jarabe de glucosa hidrogenado de alto contenido de maltosa, jarabe de glucosa hidrogenada, hidrolizado de almidón hidrogenado, poliglucitol, jarabe de poliglicitol, jarabe de sorbitol, jarabe de sorbitol ins420ii, lactitol, lactit, lactitolum, lactobiosit, lactobiositol, lactobiosiy, lactositol, maltotetraitol, maltitol, maltitol syrup, maltotritol, maltosa hidrogenada, mannitol, mannite, mannitol, d-manitol, meso-erythritol, meso eritritol, meso inositol, mezcla endulzante, modified polydextrose, neotame, neotamo, polyalcohol, polidextrosa a, polidextrosa n, polidextrosas, polidextrosas modificadas, polydextrose a, polydextrose n, poliglicitol, poliglucitol, polyglycitol, polyglycitol syrup, propilenglicol, propylene glycol, ibitol, sacarina, sacarina sodica, saccharin, sacarina de calcio, calcium saccharin, sacarina de potasio, potassium saccharin, sacarina de sodio, sodium saccharin, sal de aspartamo y acesulfamo, aspartamo-acesulfamo, aspartame-acesulfame salt, sales de calcio de leche y edulcorantes, sorbitol, sorbitol syrup, sorbitol liquid, sorbitol e 420, sorbit, 4,1,6-triclorogalactosacarosa, sorbol, Splenda, stevia, steviol glicosido, sucralosa, sucralose, tetrahidroxibutano, taumatina, thaumatin, threitol, volemitol, xylite, xylitol, xilitol, xilitol cristaline
**Codes**	E 420, E 421, E 953, E 953, E 964, E 965, E 966, E 967 ,E 968, E-420, E-421, E-953, E-953, E-964 , E-965, E-966, E-967, E-968, E420, E421, E953, E953, E964, E965 , E966, E967, E-950, E-969, E-952, E-956, E-951, E-961, E-954, E-962, E-955, E-957, E-960, E 950, E 969, E 952, E 956, E 951, E 961, E 954, E 962, E 955, E 957, E 960, E950, E969, E952, E956, E951, E961, E954, E962, E955, E957, E960, SIN-950, SIN-969, SIN-952, SIN-956, SIN-951, SIN-961, SIN-954, SIN-962, SIN-955, SIN-957, SIN-960, SIN 950, SIN 969, SIN 952, SIN 956, SIN 951, SIN 961, SIN 954, SIN 962, SIN 955, SIN 957, SIN 960, SIN950, SIN969, SIN952, SIN956, SIN951, SIN961, SIN954, SIN962, SIN955, SIN957, SIN960, SIN 420, SIN 421, SIN 953, SIN 953, SIN 964, SIN 965, SIN 966, SIN 967, SIN 968, SIN-420, SIN-421, SIN-953, SIN-953, SIN-964, SIN-965, SIN-966, SIN-967, SIN-968, SIN420, SIN421, SIN953, SIN953, SIN964, SIN965, SIN966, SIN967, SIN968.

Table S3. Number of products declaring nutrients of concern in the three collections

Category	Total (n)	Sugar n (%)	Saturated fat n (%)	Trans Fats n (%)	Sodium n (%)
**Beverages**	**29**	**29 (100)**	**13 (45)**	**10 (34)**	**29 (100)**
Juice, nectars and flavored juice drinks	7	7 (100)	1 (14)	1 (14)	7 (100)
Carbonated drinks	8	8 (100)	2 (25)	0 (0)	8 (100)
Sports drinks	3	3 (100)	1 (33)	0 (0)	3 (100)
Teas	2	2 (100)	0 (0)	0 (0)	2 (100)
Milk and Dairy drinks	6	6 (100)	6 (100)	6 (100)	6 (100)
Powdered milk flavors	3	3 (100)	3 (100)	3 (100)	3 (100)
**Foods**	**65**	**64 (98)**	**62 (95)**	**62 (95)**	**65 (100)**
Bread and Bakery Products	17	17 (100)	17 (100)	17 (100)	17 (100)
Cereals	6	6 (100)	6 (100)	6 (100)	6 (100)
Candies and Desserts	8	8 (100)	5 (63)	5 (63)	8 (100)
Prepared, Ready to Eat Meals	5	4 (80)	5 (100)	5 (100)	5 (100)
Meat and Eggs	1	1 (100)	1 (100)	1 (100)	1 (100)
Ice cream	4	4 (100)	4 (100)	4 (100)	4 (100)
Dairy Based products	4	4 (100)	4 (100)	4 (100)	4 (100)
Sauces, Spreads and Oils	11	11 (100)	11 (100)	11 (100)	11 (100)
Snacks	9	9 (100)	9 (100)	9 (100)	9 (100)
